# Diurnal Variation in Urodynamics of Rat

**DOI:** 10.1371/journal.pone.0012298

**Published:** 2010-08-19

**Authors:** Gerald M. Herrera, Andrea L. Meredith

**Affiliations:** 1 Catamount Research & Development Company, St. Albans, Vermont, United States of America; 2 Med Associates, Inc., St. Albans, Vermont, United States of America; 3 Department of Physiology, University of Maryland School of Medicine, Baltimore, Maryland, United States of America; Vanderbilt University, United States of America

## Abstract

In humans, the storage and voiding functions of the urinary bladder have a characteristic diurnal variation, with increased voiding during the day and urine storage during the night. However, in animal models, the daily functional differences in urodynamics have not been well-studied. The goal of this study was to identify key urodynamic parameters that vary between day and night. Rats were chronically instrumented with an intravesical catheter, and bladder pressure, voided volumes, and micturition frequency were measured by continuous filling cystometry during the light (inactive) or dark (active) phases of the circadian cycle. Cage activity was recorded by video during the experiment. We hypothesized that nocturnal rats entrained to a standard 12:12 light:dark cycle would show greater ambulatory activity and more frequent, smaller volume micturitions in the dark compared to the light. Rats studied during the light phase had a bladder capacity of 1.44±0.21 mL and voided every 8.2±1.2 min. Ambulatory activity was lower in the light phase, and rats slept during the recording period, awakening only to urinate. In contrast, rats studied during the dark were more active, had a lower bladder capacities (0.65±0.18 mL), and urinated more often (every 3.7±0.9 min). Average bladder pressures were not significantly different between the light and dark (13.40±2.49 and 12.19±2.85 mmHg, respectively). These results identify a day-night difference in bladder capacity and micturition frequency in chronically-instrumented nocturnal rodents that is phase-locked to the normal circadian locomotor activity rhythm of the animal. Furthermore, since it has generally been assumed that the daily hormonal regulation of renal function is a major driver of the circadian rhythm in urination, and few studies have addressed the involvement of the lower urinary tract, these results establish the bladder itself as a target for circadian regulation.

## Introduction

Daily/circadian (∼24 hr) rhythms are essential for survival and fitness in virtually all organisms. Physiological function is phase-locked to the day-night cycle to ensure optimal performance of a system at the appropriate time of day, for example, an upregulation of metabolic pathways during the period of expected food consumption [1], [2]. In humans, a clear circadian rhythm in urinary function and behavior is present [3]. Storage of urine in the bladder is predominant at night, due to increased bladder capacity, and voiding predominates during the day [4]–[7]. Urinary frequency is higher during the day and is associated with a significantly higher glomerular filtration rate (GFR) in the kidney [8]–[13]. However, the functional aspects of bladder that influence daily urination behavior have not been well-studied.

Nocturia, aberrant waking at night to void, is one disorder which would benefit from understanding how daily rhythmicity is imparted to the lower urinary tract. Nocturia is associated with a loss of the diurnal variation in GFR in some cases [8], but nocturia is a common feature of many other lower urinary disorders, such as overactive bladder (OAB), partial bladder outlet obstruction (PBOO), interstitial cystitis (IC), and is correlated with advanced age [5], [14]–[16]. Several studies have shown that relief of OAB does not necessarily resolve nocturnal voiding [14], underscoring the potentially complex interplay between bladder urodynamics and the diurnal control of urination behavior. Consequently, clinically-relevant assessment of urodynamic function and dysfunction may require intact behavioral states and necessitates the development of urodynamic recording configurations where these aspects are integrated.

In rodents, the primary genetic animal models used to characterize lower urinary tract symptoms, most studies are performed during the day. Since most lab rodent strains are nocturnal, these assessments of bladder function are performed during the animal's inactive period, when the animal would normally be sleeping. Interpretations of these data may be enhanced by a fuller understanding of the phase and daily range of voiding parameters and bladder function. The goal of this study was to identify urodynamic parameters that varied between day and night in intact, behaviorally integrated rats using continuous filling cystometry.

## Results

**Figure 1:**
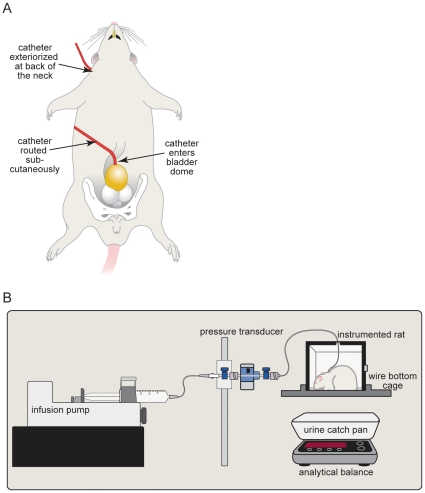
Cystometry Setup. *A:* Schematic drawing of the surgically implanted intravesical catheter into rat bladder. *B:* Schematic drawing of the cystometry test chamber, consisting of a wire bottom cage positioned above an analytical balance. A syringe pump is connected to a pressure transducer, which is connected to the intravesical catheter.

**Figure 2:**
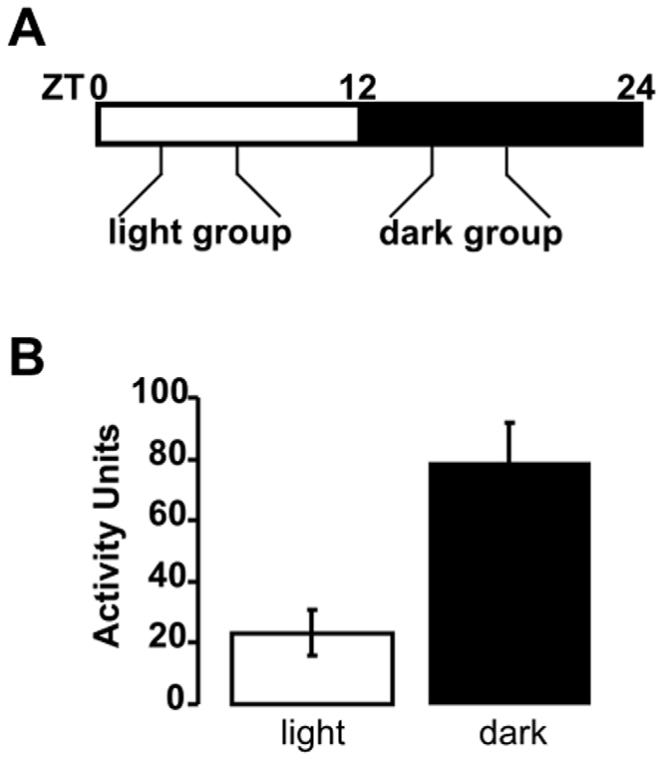
Total ambulatory activity during cystometry. *A:* CMG recordings were performed during opposite phases of the circadian cycle, 3–7 hours after lights on (ZT3-7, light group) or 3–7 hours after lights off (ZT15-19, dark group). ZT, Zeitgeber time. *B:* The average ambulatory activity during three consecutive micturition cycles from rats assayed during the light and dark phases. Dark rats showed significantly more activity than light rats (P<0.05).

Lab animal urodynamic studies present many challenges, including urine loss due to evaporation, and lack of standardization of research methods regarding filling rates, time of day of experiment, and use of anesthetized versus conscious animal models. The goal of this study was to perform *in vivo* assessments of bladder function in conscious, freely moving animals at opposite times of day, to test the hypothesis that urodynamics differ with circadian phase. Rats were divided into two groups and entrained to either a regular or reversed light:dark cycle (see Methods). Rats were surgically instrumented with an intravesical catheter for cystometry analysis (Fig. 1). After a one-week recovery period, bladder function was assayed using continuous filling cystometry. One group was assayed during the light phase, corresponding to the inactive period for rats when they would normally be sleeping. The second group was assayed during the dark phase, corresponding to the normal active period for nocturnal species. Each group underwent cystometrogram (CMG) recordings in response to continuously infused saline at the respective circadian times, ZT3-7 (light) or ZT15-19 (dark) (Fig. 2*A*). The volume of saline infused into the bladder was recorded, and rats were housed in a cage over an analytical balance to measure the voided volume during filling-voiding (micturition) cycles.

To determine whether animals exhibited behavioral patterns consistent with their circadian phase, ambulatory activity was measured from video recordings during each of the CMGs (Fig. 2*B*). Rats assayed during the dark phase exhibited higher total levels of ambulatory activity during CMG recordings (78.9±12.7 activity units, A.U.) than rats from the light period (23.2±7.5 A.U.). These data show the presence of a normal nocturnal bias to ambulatory activity after recovery from surgery and the preservation of this circadian behavior in rats undergoing continuous filling cystometry.

**Figure 3:**
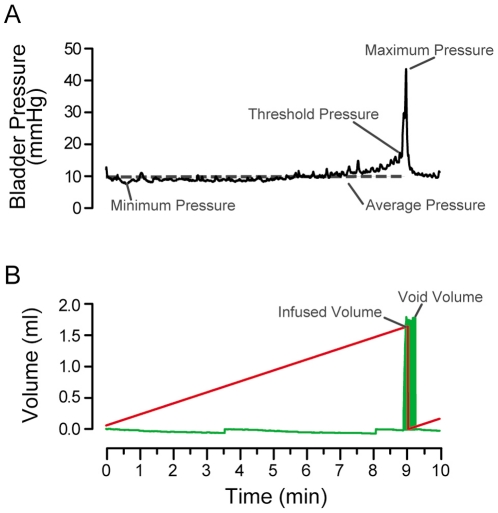
Normal filling-voiding micturition cycle. Representative cystometrogram (CMG) from a rat during the light phase in response to continuously-infused saline. Measurements were made of infused and voided volumes and bladder pressure at indicated points of the micturition cycle.

**Figure 4:**
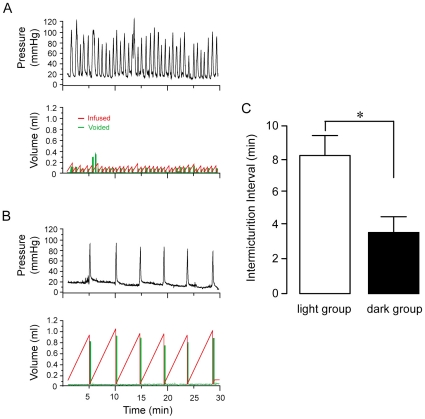
Urodynamic profile of conscious, freely moving rats during continuous infusion of saline into the bladder. *A:* Representative CMG recorded during the dark phase when rats are active. Micturition frequency is higher, and bladder capacity is reduced. *B:* Representative CMG recorded during the light phase when rats are less active or sleeping. Micturition frequency is reduced, and bladder capacity is increased. *C:* Average micturition interval is increased in light versus dark rats (P<0.05).

We hypothesized that rats would show a difference in micturition frequency between the light and dark periods of the circadian cycle. Based on the behavioral activity-rest cycle, micturition frequency was expected to be lower during the light period, when rats normally sleep. Micturition cycles were measured by CMG analysis in response to infused saline (Fig. 3). CMG recordings were comprised of simultaneous measurements of intravesical pressure, infused and voided volume during consecutive filling-voiding cycles. Both light and dark rats exhibited regular filling, followed by voiding events over the 30–90 minute recording period (Fig. 4*A*–*B*). Dark rats had a three-fold higher rate of voiding over the course of a CMG session than light rats (dark: 34±11 voids per hour and light: 10±2 voids per hour, p<0.05, Mann-Whitney Rank Sum Test). The inter-micturition interval was also significantly decreased in dark rats (dark: 3.7±0.9 min and light: 8.2±1.2 min, p<0.05) (Fig. 4*C*). These CMGs show that micturition occurrence substantially differs in rats over the circadian cycle.

**Figure 5:**
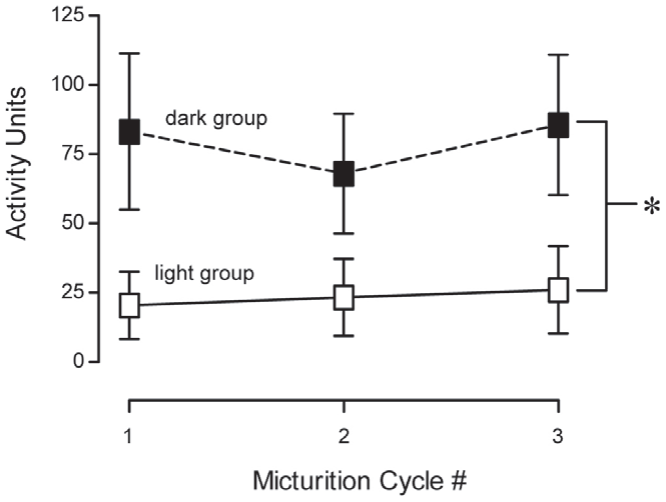
Average ambulatory activity during three consecutive micturition cycles from rats assayed during the light and dark phases. Dark rats showed significantly more activity during micturition events than light rats (P<0.05).

Behavior during individual micturition cycles was analyzed in more depth to understand how the circadian difference in micturition frequency manifested in the animal's behavior. During each individual micturition cycle, ambulatory activity was consistently higher in dark rats compared to light rats (Fig. 5). Manual scoring of behavior, based on visual observation of the video, revealed that rats studied in the dark period often exhibited vigorous exploratory behavior (score of 5) during the micturition events (average score: 2.12±0.57; Video S1). By comparison, rats from the light period were often motionless and quiescent or asleep (score of 0) throughout most of the urodynamic test, sometimes awaking only to void (0.79±0.44, p<0.05; Video S2). These data show that during cystometry under constant infusion into the bladder, rats in the light phase maintain their normal resting behavior, exhibiting increases in arousal primarily surrounding voiding events. In contrast, dark phase rats display normal exploratory behavior expected from animals in the active phase. Furthermore, the constant bladder infusion does not cause hyperactivity in either group of animals.

**Figure 6:**
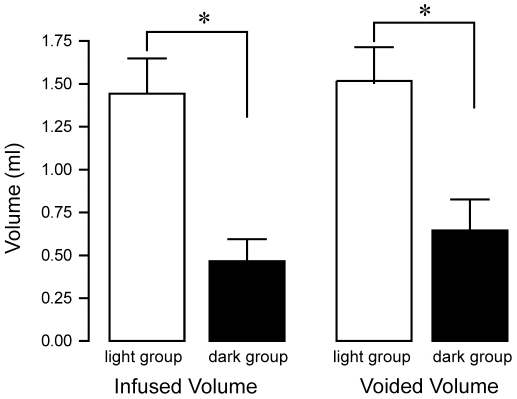
Average infused and voided volumes from micturition events. Average infused and voided volumes are reduced in dark versus light rats (P<0.05), leading to a decrease in bladder capacity (infused minus voided volume) in the dark rats group.

In order to determine the urodynamic changes that mediate the difference in micturition frequency between light and dark rats, bladder capacities and pressures were determined from CMG recordings. The filling and voiding cycle differed significantly between light and dark rats. Infused and voided volumes were about 3- and 4-fold more, respectively, in the light group (Fig. 6 and Table 1). This led to an overall increase in bladder capacity in light rats (1.44±0.21 mL) compared to dark rats (0.65±0.18 mL, p<0.05). However, average bladder pressures did not change between light and dark, nor did minimal and maximal pressures (Table 1). These data show that bladder function is geared toward storage during the rest period of the circadian cycle, and the storage capacity is reduced during the active period.

**Table 1:** Urodynamic Parameters in Light and Dark Rats.

	Light Group	Dark Group
**Infused Volume (ml)**	1.44±0.21	0.47±0.13*
**Voided Volume (ml)**	1.52±0.21	0.65±0.18*
**Intermicturition Interval (min)**	8.2±1.2	3.7±0.9*
**Minimum Pressure (mmHg)**	9.3±2.0	8.8±2.4
**Average Pressure (mmHg)**	13.4±2.5	12.1±2.9
**Threshold Pressure (mmHg)**	13.9±1.3	15.2±2.7
**Maximum Pressure (mmHg)**	68.0±10.6	52.2±10.6

Summary of urodynamic data. Mean ± SEM for CMG parameters obtained from rats tested during the light and dark phases. *P<0.05.

## Discussion

Chronically implanted catheters enable *in vivo* recordings of urodynamic parameters at different times of day and night. In this study, we show that chronically-implanted rats recorded in cystometry chambers, and subjected to constant fluid infusion into the bladder, exhibit patterns of activity consistent with the normal nocturnal behavior of the rat. A circadian difference in bladder capacity and concomitant change in micturition frequency was identified between rats in their resting versus active phases. During the light (resting) phase when animals exhibit more sleep, bladders are geared toward urine retention. In the dark (active) phase, rats show increased voiding frequency, precipitated by a decreased bladder capacity. Since baseline bladder capacities vary by 2-fold between light and dark, and micturition frequency varies by 6-fold, our data show that consideration of circadian phase is an important component to interpreting urodynamic measurements. Furthermore, a potential limitation to evaluating animal models for symptoms that mimic human dysfunction is that cystometry performed during the day (light period) in nocturnal animals evaluates the bladder in a high-capacity, quiescent state which may be differentially resistant to perturbation.

Although a clear diurnal rhythm is expressed in the micturition frequency of rodents that have not undergone a surgical procedure [17]–[19], few studies have directly addressed the daily variation in urodynamics in post-surgery, catheterized animal models. Two previous studies of the circadian profile of bladder capacity from anesthetized, chronically-implanted mice differ from the data presented here, showing a lower bladder capacity during the light phase which increases in the early portion of the dark phase [20]–[21]. Because those animals were anesthetized, it remains to be determined how the phase of the normal voiding cycle compares in rat and mouse bladders. Another study in unanesthetized, freely moving rats showed an increase in bladder capacity and micturition threshold during sleep compared to awake animals [22]. However, both measurements were performed on rats in the light phase of the circadian cycle, revealing only a difference in the urodynamics of different arousal states.

Cystometry recordings are often performed in anesthetized animals, which may affect the storage capacity of the bladder [23]–[26], but anesthetic exposure may also affect the subsequent circadian patterning of behavior after recovery [27]–[29]. Furthermore, studies in anesthetized animals cannot address the relationship between micturition and behavior. The present study shows that the normal circadian behaviors of the rat can be preserved in an experimental set-up, facilitating the usage of chronically-catheterized animals for behaviorally-integrated studies of bladder-based disorders.

The circadian variation in urodynamics depends on daily urine production, the biophysical properties of the bladder, and neural control. The diurnal variation in GFR is well-documented in humans and animals [3], [8], [30], and circadian-controlled hormone secretion is a major driver of the daily variation in urine production by the kidney [31]. However, in this study urodynamic parameters were measured in response to infused saline, removing the influences of fluid intake or urine production on cystometric measurements. Although these infused volumes are higher than normal natruesis in rat, this technique eliminates the influence of circadian variation in GFR on micturition frequency, which is a caveat to recording spontaneous events. Therefore, these recordings reflect actual day-night changes in bladder capacity, not the circadian variation in urine production. The data presented here suggest that there are multiple circadian targets involved in regulating the day-night difference in urination behavior, including the bladder itself.

The mechanism(s) of the daily change in bladder capacity is not clear. In the absence of kidney influence on the circadian variation of urodynamic function, our data are consistent with a neural or bladder-based mechanism, or a combination of the two. Tract tracing studies have identified a potential innervation chain between the brain's circadian clock, the suprachiasmatic nucleus in the hypothalamus, and the bladder [32]. Since autonomic control is also thought to play a major role in regulating the circadian variation of function in other peripheral organs, such as the heart and liver [33]–[36], it is likely that modulation of sensory and motor control of the lower urinary tract in rats contributes to the daily variation in bladder capacity and micturition frequency. Indeed, our observation that baseline bladder pressures were not different between light and dark animals could suggest that circadian regulation of bladder tone does not significantly contribute to the daily functional bladder differences. However, circadian variations in bladder biophysical properties, including excitability and contractility of bladder smooth muscle, cannot be ruled out entirely. Since the threshold bladder pressure was reached at lower filling volumes in the dark phase animals compared to the light phase animals, one possible mechanism to account for this would be daily alterations in bladder wall tone that would change the relationship between bladder volume and sensory nerve output.

In conclusion, the data presented here support the concept that the day-night difference in micturition frequency results from a daily difference in bladder capacity, independent of kidney function. Future work addressing the mechanism of the daily control of bladder function will be essential to understanding complex behaviorally-integrated disorders that result from a derangement of the normal state, such as nocturia.

## Materials and Methods

### Ethics statement

All experiments were reviewed and approved by the Institutional Animal Care and Use Committee at Med Associates, Inc. (IACUC Protocol #2005-005).

### Experimental Design

Male Sprague Dawley rats (Charles River Laboratories), ∼9 weeks old, with body weights 227–273 g were used for all experiments. Standard rat chow and water were provided ad libitum. Upon receipt of the animals from the vendor, rats were divided into two groups and entrained to 12 h:12 h light:dark cycles of opposite phases for at least two weeks. The first group was housed on a standard light:dark cycle, with lights on at 07:00 hrs and off at 19:00 hrs. After surgical implantation of the catheter and recovery (see “*Urodynamic measurements in conscious, unrestrained rats*” below), this group had cystometry performed during the first half of the light period (10:00 to 14:00, Zeitgeber time, ZT3 to ZT7). All work was performed in the light. The second group was housed on a reverse light:dark cycle, with lights on at 19:00 hrs and off at 07:00 hrs. After surgery and recovery, measurements were made from the second group during the first half of the dark period from 10:00 to 14:00, corresponding to ZT15 to ZT19. Dark animals were put into the chambers and cystometry was performed in the dark. For all animals, videography was done using near infra-red illumination (NIR-100, Med Associates, Inc.), excluding visible light with a filter installed on the camera (VID-LENS-NIR-1, Med Associates, Inc.). Numbers presented in the text are group averages ± s.e.m. Comparisons between the light and dark groups were made with 2-tailed, unpaired t-tests. The Mann-Whitney Rank Sum Test was used to compare the number of voids per hour due to an outlying data point in the dark group of rats.

### Urodynamic measurements in conscious, unrestrained rats

Regular and reversed light:dark cycle rats were anaesthetized with isoflurane (1–3% in O_2_, inhaled). Light exposure in the reversed light:dark cycle animals was 10 mins until the anesthesia took effect. A lower midline abdominal incision was made to expose the urinary bladder and a polyethylene catheter (PE-50) was inserted into the dome of the urinary bladder and secured in place using a 6-0 prolene purse string suture. The bladder catheter was sealed and routed subcutaneously to the back of the neck, where it was coiled and stored in a skin pouch. One week later, the bladder catheter was exteriorized and opened. The animal was placed in a Small Animal Cystometry Lab Station (Catamount Research and Development Company, St. Albans, VT) for urodynamic measurements. The catheter was connected to one port of a pressure transducer. The other port of the pressure transducer was connected to a syringe pump. Sterile isotonic saline (0.9% NaCl; room temperature) was continuously infused into the bladder at a rate of 175 µl min^−1^ at room temperature. An analytical balance beneath the wire-bottom animal cage measured the amount of urine voided during continuous cystometry.

Cystometrograms (CMGs) were measured over a period of 30–90 minutes, simultaneously recording intravesical (bladder) pressure, infused and voided bladder volumes using MED-CMG software (Catamount Research & Development Company). A single CMG was defined as the simultaneous recording of intravesical pressure, infused volume and voided volume during a single filling-voiding (micturition) cycle. Micturition cycles were recorded until at least 3 consecutive CMGs were reproducible (bladder capacities within 20±10% of each other). Data analysis was performed by averaging three to four consecutive CMGs. Bladder capacity was measured as the amount of saline infused into the bladder at the time when micturition commenced. Residual volume for each CMG was measured as the voided volume subtracted from the infused volume. Minimum pressure was the lowest pressure observed during the filling phase of the CMG, and peak pressure was the maximum pressure observed during the CMG. Threshold pressure was the intravesical pressure just before initiation of micturition. At the end of the experiment, the rats were euthanized with pentobarbital sodium (195 mg/kg ip) followed by decapitation.

### Ambulatory activity measurements

Animals were acclimated to the cystometry chamber for 5 to 10 min before recordings were started. A digital video camera (VID-CAM-MONO2A, MED Associates) and Video Monitor Software (Med Associates, Inc.) recorded the animal's activity during the experiment using near-infrared illumination. A custom analysis program was used to quantify the amount of ambulatory activity for each animal based on the amount of movement detected frame-to-frame in the video recording. Motion was quantified by subtracting the video signal of a reference segment (empty cage) from the video of the animal in the cage during cystometry recordings. The resulting motion, in arbitrary ambulatory units (A.U.), was used as an index of locomotor activity. Manual scoring of behavior was performed blind using a scale of 0 (asleep) to 5 (intense activity, exploring chamber).

## Supporting Information

Video S1 Behavioral activity of a rat from the dark phase group in the cystometry chamber. Voiding events noted.
(5.23 MB MOV)

Video S2 Behavioral activity of a rat from the light phase group in the cystometry chamber. Voiding events noted.
(5.14 MB MOV)

## References

[1] Reppert SM, Weaver DR (2002). Coordination of circadian timing in mammals.. Nature.

[2] Green CB, Takahashi JS, Bass J (2008). The meter of metabolism.. Cell.

[3] Mills JN (1951). Diurnal rhythm in urine flow.. J Physiol.

[4] Van Hoeck K, Bael A, Lax H, Hirche H, van Gool JD (2007). Circadian variation of voided volume in normal school-age children.. Eur J Pediatr.

[5] Ku JH, Lim DJ, Byun SS, Paick JS, Oh SJ (2004). Nocturia in patients with lower urinary tract symptoms: association with diurnal voiding patterns.. BJU Int.

[6] Weiss JP, Blaivas JG (2003). Nocturia.. Curr Urol Rep.

[7] Chamberlain PF, Manning FA, Morrison I, Lange IR (1984). Circadian rhythm in bladder volumes in the term human fetus.. Obstet Gynecol.

[8] De Guchtenaere A, Vande Walle C, Van Sintjan P, Raes A, Donckerwolcke R (2007). Nocturnal polyuria is related to absent circadian rhythm of glomerular filtration rate.. J Urol.

[9] Koopman MG, Krediet RT, Arisz L (1985). Circadian rhythms and the kidney. A review.. Neth J Med.

[10] Buijsen JG, van Acker BA, Koomen GC, Koopman MG, Arisz L (1994). Circadian rhythm of glomerular filtration rate in patients after kidney transplantation.. Nephrol Dial Transplant.

[11] van Acker BA, Koomen GC, Koopman MG, Krediet RT, Arisz L (1992). Discrepancy between circadian rhythms of inulin and creatinine clearance.. J Lab Clin Med.

[12] Goldman R (1951). Studies in diurnal variation of water and electrolyte excretion; nocturnal diuresis of water and sodium in congestive cardiac failure and cirrhosis of the liver.. J Clin Invest.

[13] Wesson LG (1964). Electrolyte Excretion in Relation to Diurnal Cycles of Renal Function.. Medicine (Baltimore).

[14] Drake NL, Flynn MK, Romero AA, Weidner AC, Amundsen CL (2005). Nocturnal polyuria in women with overactive bladder symptoms and nocturia.. Am J Obstet Gynecol.

[15] Weiss JP, Blaivas JG (2002). Nocturnal polyuria versus overactive bladder in nocturia.. Urology.

[16] Kelada E, Jones A (2007). Interstitial cystitis.. Arch Gynecol Obstet.

[17] Schmidt F, Yoshimura Y, Ni RX, Kneesel S, Constantinou CE (2001). Influence of gender on the diurnal variation of urine production and micturition characteristics of the rat. Neurourol.. Urodynamics.

[18] Longhurst PA, Eika B, Leggett RE, Levin RM (1992). Comparison of urinary bladder function in 6 and 24 month male and female rats.. J Urol.

[19] Stewart FA, Michael BD, Denekamp J (1978). Late radiation damage in the mouse bladder as measured by increased urination frequency.. Radiation Res.

[20] Dorr W (1992). Cystometry in mice--influence of bladder filling rate and circadian variations in bladder compliance.. J Urol.

[21] Dorr W, Kraft M (1997). Effects of ageing and X-irradiation on the diurnal rhythm of mouse urinary bladder capacity.. Urol Int.

[22] Kiddoo DA, Valentino RJ, Zderic S, Ganesh A, Leiser SC (2006). Impact of state of arousal and stress neuropeptides on urodynamic function in freely moving rats.. Am J Physiol Regul Integr Comp Physiol.

[23] Oliver JE, Young WO (1973). Evaluation of pharmacologic agents for restraint in cystometry in the dog and cat.. Am J Vet Res.

[24] Smith PP, Hurtado E, Smith CP, Boone TB, Somogyi GT (2008). Comparison of cystometric methods in female rats.. Neurourol Urodyn.

[25] Matsuura S, Downie JW (2000). Effect of anesthetics on reflex micturition in the chronic cannula-implanted rat.. Neurourol Urodyn.

[26] Ghoniem GM, Shoukry MS, Monga M (1996). Effects of anesthesia on urodynamic studies in the primate model.. J Urol.

[27] Prudian F, Gantenbein M, Pelissier AL, Attolini L, Bruguerolle B (1997). Daily rhythms of heart rate, temperature and locomotor activity are modified by anaesthetics in rats: a telemetric study.. Naunyn Schmiedebergs Arch Pharmacol.

[28] Dispersyn G, Pain L, Touitou Y (2009). Circadian disruption of body core temperature and rest-activity rhythms after general (propofol) anesthesia in rats.. Anesthesiology.

[29] Colwell CS, Kaufman CM, Menaker M, Ralph MR (1993). Light-induced phase shifts and Fos expression in the hamster circadian system: the effects of anesthetics.. J Biol Rhythms.

[30] Shirley DG, Walter SJ, Zewde T (1989). Measurement of renal function in unrestrained conscious rats.. J Physiol.

[31] Zuber AM, Centeno G, Pradervand S, Nikolaeva S, Maquelin L (2009). Molecular clock is involved in predictive circadian adjustment of renal function.. Proc Natl Acad Sci U S A.

[32] Sly DJ, Colvill L, McKinley MJ, Oldfield BJ (1999). Identification of neural projections from the forebrain to the kidney, using the virus pseudorabies.. J Auton Nerv Syst.

[33] Bartness TJ, Song CK, Demas GE (2001). SCN efferents to peripheral tissues: implications for biological rhythms.. J Biol Rhythms.

[34] Kalsbeek A, La Fleur S, Van Heijningen C, Buijs RM (2004). Suprachiasmatic GABAergic inputs to the paraventricular nucleus control plasma glucose concentrations in the rat via sympathetic innervation of the liver.. J Neurosci.

[35] Oosting J, Struijker-Boudier HA, Janssen BJ (1997). Autonomic control of ultradian and circadian rhythms of blood pressure, heart rate, and baroreflex sensitivity in spontaneously hypertensive rats.. J Hypertens.

[36] Kalsbeek A, Perreau-Lenz S, Buijs RM (2006). A network of (autonomic) clock outputs.. Chronobiol Int.

